# Informed Consent in Complementary and Alternative Medicine

**DOI:** 10.1093/ecam/nep032

**Published:** 2010-10-19

**Authors:** Opher Caspi, Tamar Shalom, Joshua Holexa

**Affiliations:** ^1^Integrative Medicine Unit, Rabin Medical Center and the Tel-Aviv University, Petah Tikva 49100, Israel; ^2^Department of Health System Management, Ben-Gurion University, Israel; ^3^Department of Emergency Medicine, University Medical Center, Tucson, AZ, USA

## Abstract

The objective of this study was to examine complementary and alternative medicine (CAM) practitioners' (i) attitudes toward informed consent and (ii) to assess whether standards of practice exist with respect to informed consent, and what these standards look like. The design and setting of the study constituted face-to-face qualitative interviews with 28 non-MD, community-based providers representing 11 different CAM therapeutic modalities. It was found that there is great deal of variability with respect to the informed consent process in CAM across providers and modalities. No unique profession-based patterns were identified. The content analysis yielded five major categories related to (i) general attitude towards the informed consent process, (ii) type and amount of information exchange during that process, (iii) disclosure of risks, (iv) discussions of alternatives, and (v) potential benefits. There is a widespread lack of standards with respect to the practice of informed consent across a broad range of CAM modalities. Addressing this problem requires concerted and systematic educational, ethical and judicial remedial actions. Informed consent, which is often viewed as a pervasive obligation is medicine, must be reshaped to have therapeutic value. Acknowledging current conceptions and misconception surrounding the practice of informed consent may help to bring about this change. More translational research is needed to guide this process.

## 1. Introduction

“I don't want to see forms of informed consent because [if that] happens, insurance [companies] will squeeze the life out of herbal medicine.”

C.K. (a herbalist)

Informed consent (IC) is an integral and imperative component of medical practice. It has existed in myriad forms for years, yet there remain few commonly accepted norms or clear criteria dictating the type, scope and quality of information needed to obtain it in day-to-day practice. Likewise, very few guidelines exist for how much discussion is adequate to make a complete informed decision in clinical scenarios of varying complexity [1].

While the conventional medical establishment has taken concerted steps (in the form of lawsuits and subsequent policy changes) to outline procedures pertaining to IC [2], comprehensive guidelines enumerating, defining, and explaining all aspects of this important component of medicine remain insufficient [3]. This suboptimal reality might ostensibly be even worse in the emergent field of complimentary and alternative medicine (CAM) where a recent study illustrates troubling lack of standards and uniformity of practice with respect to the methods of IC across different modalities [4].

Concerns exist that IC in CAM might be even more complex than in conventional medicine (allopathy) considering that it encompasses multiple modalities to choose from, is usually provided by non-MD practitioners who might differ greatly one from another, and it uses a language that is not always clear to patients (e.g., needling in acupuncture). This creates an environment that, in sharp contrast to CAM's patient-friendly image, may, at least in theory, inadvertently compromise patient autonomy, impair compliance with treatment, and even have negative consequences on therapeutic outcomes.

Despite this obvious divergence from conventional biomedicine it remains unknown whether IC procedures in CAM should, or do indeed differ from allopathy. To begin to examine this important aspect of patient-centered healthcare, we conducted an exploratory qualitative study with a cohort of non-MD practitioners of various CAM modalities to determine the key issues associated with the process of IC in CAM. Specifically, we were interested in the following questions.

- What constitutes “informed” in the IC process? In other words, what information do practitioners offer to patients in clinical scenarios to “inform” them, and how and when is this exchange deemed sufficient?
- Whether the scope of IC and its procedures are contextually defined? That is, whether the nature of CAM therapies creates an environment in which the process of IC would differ from allopathic IC processes?
- What are the perceived barriers and facilitators toward a more appropriate practice of IC within the context of CAM?

## 2. Methods

For the purposes of this study we followed the operational definition and classification system of CAM put forward by the NIH National Center for Complementary and Alternative Medicine (NCCAM), “a group of diverse medical and healthcare systems, practices, and products that are not presently considered to be part of conventional medicine [5].” Since the study's primary focus was on IC, only provider-administered CAM modalities, such as acupuncture, naturopathy, osteopathy and so forth. were considered. Following other national CAM utilization surveys [6], self-administered CAM practices, such as daily supplemental vitamin use were excluded. The University of Arizona Institutional Review Board pre-approved all study's procedures and forms.

### 2.1. Participants

Locally based non-MD CAM practitioners in the Tucson, Arizona area were identified using the yellow pages as well as personal contacts. All were licensed practitioners with both basic and advanced (post-graduate) professional training, and practiced in solo, community-based settings for many years. Once identified, they were contacted by phone to present the aim of the study, and to solicit participation. Participation consisted of a one-hour tape recorded structured interview. If consent was given, a list of questions relating to various aspects of the practice of IC was faxed to them (Supplementary Table1). Practitioners were asked to reflect on these questions prior to the meeting in order to facilitate a more fruitful interview. The practitioners were asked to read and sign a subject consent form and a subject authorization form prior to the interview.

### 2.2. Interviews

Personal interviews were conducted at the provider's office at an agreed upon and convenient time. Questions and language used to conduct the interviews was intentionally open-ended, broad, and non-directive. The interviewers (OC and JH) did not provide any biasing information or suggestions as to their personal views of the practice of IC. Instead, they encouraged subjects to elaborate and clarify key points, and inquire further about any other concerns or comments subjects might have had with respect to the practice of IC in CAM. The goal was to encourage practitioners to reflect on (i) their own personal experiences with the practice of IC, (ii) their personal viewpoint of what IC should consists of, and (iii) their beliefs about how IC should be conducted. To learn more about the norms of IC in CAM, we also asked practitioners to provide us with copies of their IC forms (if they had any) or any other documents that they use to consent patients.

### 2.3. Data Analysis

All interviews were tape-recorded, and inductive content analysis was used as the primary method to analyze the data collected. This method, as developed within the social sciences and linguistics, involves the process of inductive data reduction to distill the most important or essential domains of experience from the words of the subjects [7, 8]. Rather than try to fit the responses into preconceived categories developed by the researchers, inductive content analysis allows categories to “emerge” from the perspective of the participants. This characteristic of inductive content analysis is particularly helpful because, as emphasized above, the primary goal of this study was to explore various providers' perceptions and experiences with respect to the practice of IC in CAM.

Words and word phrases related to each of the research questions were identified from the data recorded, first independently and subsequently collectively, by all researchers (O.C., T.S. and J.H.). These data units were first identified using participants' phrases, and subsequently reduced to abstract-theoretical codes by combining similarly stated coded categories. Once the abstract coding units were identified, data were again reduced and categories developed to reflect broadly the responses to the main exploratory research questions. Common methods were used to assure scientific rigor, trustworthiness, dependability, and confirmability of the analytic process [8, 9].

## 3. Results

A total of 28 non-MD CAM practitioners (15 males; 13 females) representing 11 commonly used CAM modalities were interviewed (six acupuncturists, four naturopaths, three homeopaths, three osteopaths, two hypnotherapists, two herbalists, two massage therapists, two chiropractors, two energy medicine providers, and two shamans).

Very few consistent standards, approaches, or attitudes were found with respect to the IC process in CAM across providers and modalities. That is, in addition to the paucity of standards in CAM itself, there existed no unique profession-based patterns either. Rather, CAM practitioners seem to represent their own opinions or preferences and not profession-based standards, perhaps because there are none [4].

The content analysis yielded five major categories related to (i) general attitude towards the IC process, (ii) type, structure and amount of information exchange during that process, (iii) disclosure of risks, (iv) potential benefits, and (v) discussions of alternatives.

**General Attitude towards the IC Process**

Few practitioners welcomed the IC process as a means to empower and educate patients, whereas many more providers regarded it merely as a legal or administrative nuisance, and had concerns regarding liability issues. Also, some providers did not welcome the IC process at all, either as an educational opportunity or as a legal/ethical requirement (Supplementary Table2).

CAM practitioners varied greatly with respect to the amount and type of information they regard as necessary to the IC process. Some providers make sure that patients have access to all material information, and value completeness of information, while others elect what information to share and what not based predominantly on questions the patients bring up and a perception that patients might not want to know everything (Supplementary Table3).

Many interviewees expressed explicit concerns that, while acknowledging the importance of disclosing risks to patients as part of the IC process, elaborating on risks too much might actually be detrimental to the patients' healing response. Many more practitioners, on the other hand, disclose risks selectively, based either on how common they are or how serious the consequences might be. Another interesting theme that emerged was providers' perception of uncertainty with respect to the body of knowledge about risks (Supplementary Table4).

**Potential Benefits**

The community-based practice setting of our cohort creates an environment in which patients have to pre select a clinic and a provider, and proactively make an appointment; rather than being referred there by healthcare insurance. Many of our interviewees tailor the information and potential benefits of treatment they discuss with their patients in light of the self-directed and educated nature of their clientele (Supplementary Table5).

Two concerns have been raised with respect to the discussion of alternatives—knowledge and attitude. Because of the diversity of both CAM and allopathy, providers might not be aware and knowledgeable enough regarding all languages in the “tower of Babel” of medicine [10]. In addition, because they are invested in a specific modality of CAM, they might have different styles of communication and tone when they discuss alternatives, or may simply to reticent to discuss alternatives because it is simply not good business (Supplementary Table6).

## 4. Discussion

CAM is an umbrella term covering a diverse array of healing modalities [5]. Although CAM may remain marginal as to its place in the current healthcare system, the robust consumer movement towards these therapeutic options and its subsequent economic viability has thrust CAM into the mainstream [11]. Discussing and informing patients of these therapeutic options, whether driven by patients in conventional settings or offered by practitioners in CAM settings, is increasingly becoming part and parcel to standard medical care [12, 13]. This leads ostensibly to two fundamental questions: how is IC currently accomplished in the CAM setting? And is it adequate? Our approach was to attempt to understand IC as a process, as opposed to a static procedure or simple doctrine. Our questions focused on issues above and beyond the legal document and formal consent forms. We are not suggesting that these forms do not have a place in medicine, or carry little importance, however to understand how CAM practitioners can enable and encourage patients make decisions regarding their healthcare, we need to look beyond the paperwork.

The core of IC, according to the American Medical Association is “the patient's right of self-decision [which] can be effectively exercised only if the patient possesses enough information to enable an informed choice” [14]. But what constitutes information, and when it is deemed sufficient are not entirely clear. Although it is unanimously agreed that the practice of IC should at least include a discussion of three basic elements, namely risks, benefits and alternatives; the meaning, extent, depth and ratio that these three aspects occupy in the process of IC is not well defined [2]. This is perhaps why the practice of IC varies considerably not only within allopathy and CAM, but also across countries [15].

What was apparent from our interviews with CAM practitioners was their ambivalence towards the practice of IC. Practitioners often insinuated that the IC procedure is an obligatory burden with its roots in allopathic medicine, which they strive to separate themselves from, and does little if anything to truly educate the patients and involve them in the decision making process. Very few CAM practitioners had any formal IC forms. The reasons varied, but most claimed that they were not overly concerned with issues of liability because of their impression that patients who seek CAM therapies tend to be a less litigious and more informed cohort as compared to patients who rely solely on allopathy [16].

Similar to previous reports, most participants perceived the IC procedure as disruptive to building a therapeutic alliance [17, 18]. They felt that the IC forms get in the way of forming relationships, and that this formal doctrine is just “another oppressive clinical form [that] I don't even think they [patients] read.” Some practitioners even claimed that they try not to put too much in writing, because patients “… tend to internalize the negatives more readily.” This practice has obvious positive and negative implications. Considering the fearful tone of many IC doctrines that are saturated solely with risks involved of the proposed treatment, internalization can be a major difficulty—one that could lead to a nocebo effect [19]. However, if this same form was balanced with positive effects of the proposed treatment there may be an opposing belief system activated producing a placebo effect [20, 21]. There are indeed therapeutic ramifications of IC processes when one considers expectation and belief as viable factors affecting health outcomes [22, 23].

The few CAM practitioners who did utilize some formal IC doctrine did so primarily as an inoculation against possible litigation [24]. The irony is that those practitioners who did employ strict, formal consenting documents conceded that consents do little to truly protect them from any liability issues. For most participants there was little distinction between this legal aspect of IC and the ethical aspect of IC, that is, promoting autonomy and patient involvement in the decision-making process. Indeed, Diamond [25] and Ernst and Cohen [17] emphasized that the completion of a standardized consent form does not constitute consent itself; it is merely evidence that consent has been given. In other words, it does not free the practitioner from providing all the necessary information the patient may require to arrive at an informed decision. In daily practice, IC is often formulaic, authoritarian, and bureaucratic. It does not fulfill its role of stimulating conversation and dialogue between patient and practitioner.

Regardless of the presence or absence of a formal IC doctrine, all practitioners that we interviewed engaged in some sort of process of informing patients and garnering consent. However, views were incredibly divergent on what this process should encompass and how robust it should be. When prompted, all practitioners were proponents of full disclosure, often considered sufficient when the proxies' procedure/alternatives/risk were thoroughly discussed. However, when discussing the global picture of information sharing, a common theme emerged: many of the interviewees believed that information sharing (packaged as IC) is not something that can or should be mandated. Rather they thought that their main duty is to respond to patients' questions. In that way, only information that is material in bringing patients to a level of comfort and acceptance with which to begin treatment is discussed. Indeed, recent research confirmed that different patients need different information in order to make both CAM [26] and allopathic [27] related healthcare decisions.

Of all the information that should be present in IC discussions, risk has become most prevalent and is the cornerstone of this process [27]. An upside to most CAM modalities, and one reason patients are choosing this mode of treatment, is that therapies are usually non-toxic, often non-invasive and hence in the patients mind, relatively risk free [28]. Indeed, most CAM practitioners interviewed echoed the same sentiment—that the therapies they administer are so benign, there are no serious risks; and harmful outcomes could have only been caused by incompetent providers [29]. One practitioner interviewed claimed “You have to be an idiot—or actually try—to hurt someone using acupuncture. But I still tell patients [about risks] to cover my legal ass.” Another practitioner stated, “I have supreme confidence in what I do. If you give people ideas [about risks and negative outcomes] it may turn them off. I don't give people risks unless it is totally warranted and it is a risk to them.” Provider competence appears to be linked to risk intensity and prevalence in CAM modalities, at least in the minds of the providers themselves. Those practitioners that do share all risks with their patients admitted that they do so using qualifiers for example, “this could happen, but …” “it has never happened to me …”, “his is a possibility, however it has never occurred …”, and so forth, so as to tone down possible nocebo effects.

Discussion of risk disclosure with practitioners produced the most variance of any topic covered. Perhaps it is due to the abstract notion of risk in the context of CAM therapies. For example, a “risk” in hypnotherapy or energy medicine may constitute the liberation of uncomfortable emotions. A practitioner in mind-body medicine offered this view on the process of IC: “I don't tell anybody anything because you plant a seed, you contaminate their process and you get a false positive,” he went on to say that he, “will answer any questions they have—that's part of the process. But I won't volunteer information that would impede their process.”

But, as the PAR (procedure/alternatives/risk) acronym suggests, IC is not just about discussion of risks. Many people who choose to employ certain CAM modalities do so volitionally. They often have some background information on the chosen modalities, are casually familiar with the tools and techniques involved, have heard testimonials and other encouraging advice and commence treatment with a belief and an expectation that it will be of benefit to them. Worldwide surveys of CAM consumers find them to be a more empowered, educated and affluent cohort [30]. As a result many practitioners aware of this fact assume that patients may have “done their homework” prior to entering the office. For example, a recent position paper on the practice of IC in acupuncture states, “… [Informed] consent is assumed by the fact that the patient has turned up at the clinic and undresses in preparation for treatment” [31].

While most practitioners claim that knowledgeable patients are a blessing, there are situations where expectations can be unrealistic to the extent that this may pose ethical problems (e.g., false hope in incurable conditions). Also of considerable worry might be patients with unreasonable expectations based on inaccurate data and fallacious information. Indeed, many interviewees admitted that often times IC discussions are spent re-educating patients and molding expectations to be more reasonable. In addition many CAM practitioners claim they are continually proving and debunking aspects of the therapy they offer and other therapies as well as making sure to establish themselves as credible practitioners. In our cohort of interviewees this emerged as especially important for the CAM therapies that are unlicensed.

CAM Practitioners (outside of naturopaths) viewed their respective disciplines as “specialties” with no need to discuss other alternatives in the realm of CAM or allopathy unless they felt their patient was presenting a condition that was untreatable by the therapeutic tools they had to offer and/or was out of their scope of practice. For example, all acupuncturists interviewed said they would immediately refer patients with breast cancer to a board certified oncologist, or be sure that they were getting treatment by conventional means before they began giving them acupuncture sessions to support the immune system, assuage nausea and relieve discomfort associated with chemotherapy. However, no acupuncturist would offer their patient any alternatives if they came to him complaining of headaches, because headaches are a condition that can be mitigated by acupuncture [32]. Most practitioners felt that there is no need to discuss alternatives, CAM or conventional, because it is simply unnecessary for two reasons, (i) as stated by one practitioner, “If a patient comes to see me for a specific treatment, this is the treatment they desire, the decision has been made.” In other words, as mentioned earlier, patients choose to pursue a specific CAM treatment because they have procured information germane to the chosen treatment and their current complaint; and (ii) Discussion of alternatives is not pragmatic from a business standpoint. If acupuncture can treat headaches just as well as medicine, why mention the latter? This latter argument is admittedly a little bit worrisome from the IC practice standpoint since it means that patients are not deliberately informed about alternatives (the A in the PAR acronym). Also a failure to discuss alternatives may lead to a delay or withholding of a more beneficial treatment (CAM or allopathic) for a more serious disease. This could have devastating and far-reaching consequences.

Moreover, practitioners admitted that they would only offer alternatives after a treatment they prescribed failed, or worked less than optimally. However most practitioners went on to say that giving advice, recommendations, or alternatives made them uncomfortable because it was offering information they were unfamiliar with; information that was out of their domain. Herein lies a dilemma. While alternatives would be discussed under certain circumstances, that is, when prompted by questions, it was unclear how they would be discussed fairly and objectively if practitioners were uncomfortable doing so. This is, perhaps why in the end practitioners concurred that providing alternatives is the principle job of the primary care physician (MD, DO and ND). They are the ones who “… should provide options and strive toward patient decision—this is not my job. If the decision is to try [acupuncture] they come to my office and consent and decision are implied.”

Another piece of IC discussions, and one that often gets overlooked is benefits. As with other aspects of the process, most practitioners seemed to carry unwarranted assumptions. As one practitioner claimed, “… benefits are known—why else would a patient choose to come?” When practitioners were asked about describing benefits to instill a positive expectation in treatment, opinions were incongruent. Some were adamant about the “specific” efficacy of the treatment they provide, and boldly state belief plays no role (the so called “non-specific effects”) [33]. Others took a more conservative stance saying that if one discloses, “… too many benefits, patients may perceive therapy as a magic bullet and not do their part.” According to many practitioners, disclosing too many benefits creates an environment that may undermine patient responsibility and hamper patient empowerment. Words were chosen carefully when describing benefits: “I tell patient what I believe would happen if they did certain things. But I never promise an outcome.” Another practitioner talked about goals for treatment as opposed to benefits saying that he attempts to, “… shape expectation by setting goals. [This] lays a groundwork for success but does not guarantee success itself.” Other were not as concerned about the ramifications of discussing benefits claiming that, “Anyone who does not thoroughly go over benefits is severely underutilizing a powerful therapeutic tool.” Indeed, recent research into the power of belief and patient-doctor communication confirms the potency of this therapeutic tool [34, 35].

When understanding the process of IC it is important to know what CAM practitioners believe are elements that act as barriers to creating an environment where a fruitful exchange of information can take place, as well elements that encourage this process. To truly educate patients, thoroughly describe risks associated with treatment, as well as the benefits and possible alternatives takes a significant amount of time; time that few practitioners are willing to commit. If shared decision-making emerging from ongoing process of discussion and communication is how IC would best serve the patient and the practitioner, a shift in the way many of the practitioners practice would need to take place. This was the first word out of almost every practitioner's mouth when asked about barriers to the process of IC: time. Other barriers mentioned were: the view that IC is solely there for legal protection, comprehensive IC puts patients on info overload and becomes too complex, or false information may be obtained by patients and contaminate expectations. However, initial information, when in the right form, was also said to facilitate IC process. Also beneficial was patient trust in the practitioner and the treatment, helped by framing therapy as “goal driven work” and word of mouth referrals. Practitioners say patients' eagerness and interest in these therapies expedites and enhances the practice of IC. All the aspects were of great value in not only facilitating the process, but also instilling a powerful belief component that helps shape the therapeutic value of IC.

Few things were recognized as educational approaches to improve the process and practice of IC. What surfaced was the infrequency with which this topic was discussed. Very few practitioners had any educational experience with ethics courses or risk management. Just the fact of talking about IC worked to bring attention and concern to this much maligned and often overlooked aspect of patient-centered healthcare. Many practitioners expressed the desire to continue with discussions on this topic with a small group of their peers, to learn how other practitioners approach the practice of IC, gaining perspective and raising awareness. Other remedial approaches suggested were to utilize literature, video, internet, and other multimedia tools to pre-educate patients about risks, benefits and alternatives.

## 5. Conclusions and Implications

We believe that an appropriate goal is to ultimately see IC as part of the therapeutic relationship, rather than merely as a legal obligation. IC should be a process of negotiation or a discussion intended to produce an agreement [36–40]. This is an important shift in the perception of the doctor-patient relationship and a critical evolution from the paradigm of medicine as a paternalistic endeavor [18]. A common theme that emerged was that most practitioners supported full disclosure, but few willingly offered it. It was agreed that disclosing risks associated with treatment could be seen as a possible barrier to the healing process. However, doubts and uncertainties from the patients' perspective regarding the intervention and alternatives pose just as formidable barriers. Understood in this way, full disclosure is warranted and welcomed as a way to disclose risk, explain benefits and allay fears in order to come to a mutual acceptance and comfort with the proposed modality allowing for full informed decision making. This approach is congruent with the physicians need to act in beneficence as well as the patients desire to respect their autonomy.

There is a growing concern that IC is a unidirectional dutiful disclosure of risks, benefits and alternative, or as mentioned by some “formulaic, authoritarian, and bureaucratic” [17]. It has evolved to discourage patients from actively participating in decision making processes relative to their own health and well-being. Acknowledging current conceptions and misconception surrounding the practice of IC may help to bring about a change. One that seeks to fully involve the patient in all aspects of care; to respect and promote patient autonomy, to shift the current paradigm of IC from informed coercion, the direction it is apparently heading, to *informed decision*. Shaping the “event model” of IC, that as a simple procedure culminating in signature on a disclaimer, to an ongoing process involving a partnership between patient and practitioner where negotiation and communication is what prevails [3, 41, 42]. Moreover, informed and empowered patients have been shown to have more positive outcomes, to be more compliant, and to be less likely to take legal action in the instance something should go wrong [16, 20, 43, 44]. Ironically this is the primary reason IC exists today, however malpractice lawsuits continue to rise, subsequently so does malpractice insurance [45]. We believe it is time to take a fresh look at an old concept. The current IC doctrine must change.

We therefore call for a well-concerted effort in translational research using much larger, international, and trans-cultural cohorts of practitioners and patients to model common questions patients ask and explore how they are answered, and with what consistency. A translational research program in this area will explore key questions such as how are patients understanding/interpreting what they are being told? What are the pitfalls in assuming that patients are already informed and understand key concepts and terms used by practitioners? Is there a role for websites or other decision-aids that provide this information and are approved by CAM practitioner organizations? and so forth. IC, which is often viewed as a pervasive obligation is medicine, can be shaped to have therapeutic value. Can the CAM community stand up to the task? We are certain it can!

## Supplementary Data

Supplementary Data are available at *ECAM* Online.

## Funding

Short-term student fellowship funded by the National Center for Complementary and Alternative Medicine (NCCAM) at the NIH (grant T32 AT001287-01) (to J.H.).

## Supplementary Material

Table 1: List of questions for providers of complementary and alternative medicine.
Table 2: General attitude towards the informed consent process.
Table 3: Type, structure and amount of information exchange during the IC process.
Table 4: Disclosure of risks.
Table 5: Potential benefits.
Table 6: Discussions of alternatives.
