# Supplementary material for: Informed Consent in Complementary and Alternative Medicine
Source: Evid Based Complement Alternat Med. 2010 Oct 19;2011:170793. doi: 10.1093/ecam/nep032 (PMC3146982; doi:10.1093/ecam/nep032)
Supplement: Supplementary file 1 — Table 1: List of questions for providers of complementary and alternative medicine. Table 2: General attitude towards the informed consent process. Table 3: Type, structure and amount of information exchange during the IC process. Table 4: Disclosure of risks. Table 5: Potential benefits. Table 6: Discussions of alternatives. [file 170793.f1.pdf]

Supplementary Tables

Table 1: List of questions for providers of complementary and alternative medicine

1. What does the term “informed consent” mean to you?
2. Tell me your experience with informed consent.
3. What qualities make informed consent really informed?
4. What do you think are the patients’ expectation regarding the amount, type, scope, and quality of information offered by you concerning their ailment?
5. What do you think is the sufficient amount, type, scope, and quality of information you can offer?
6. What do you believe is the most important piece of information a physician can offer a patient in the consensual process?
7. What are the upsides of informed consent?
8. Does informed consent have any downsides?
9. Considering the broad definition of informed consent, how qualified are you in facilitating this process?
10. Did you receive any formal training on informed consent?
11. Would you be interested in getting further education that focuses on multiple aspects of informed consent for providers?
12. What do you think this education should be like?

Table 2. General Attitude Towards The Informed Consent Process

Theme	Example
IC process as a means to empower and educate patients.	*"For me it's informing the patient about the procedure, what I do and make sure it's okay with him. I educate the patient"*
IC process as a legal or administrative nuisance.	*"I'm not seeing it like gaining something with the patient; it's only [an] administrative task... I called it Informed Risk and Consent"*
IC process and liability.	*"The only thing I do the first [visit] is making sure they understand that I'm not a general practitioner. A lawyer told me to do that. I make sure that they have been at family physician, and if not I refer them."*
IC not welcomed at all.	*"I really believe that people can make informed decisions and it's not my responsibility…I don't want to see forms of informed consent because [if that] happens, insurance [companies] will squeeze the life out of herbal medicine."*

Table 3. Type, Structure and Amount of Information Exchange During the IC Process

Theme	Example
IC process is driven by appreciation for completeness of information.	*"I tell the patients about risks and benefits, [and] answer their questions. I give them places where they can get more information and give them alternatives. I give them all information they need to make [an informed] decision… I use everything. The more information or education the patient has is better. Motivation leads to expectation. I can't educate them well enough and the more I can explain [to] them the better. There is no situation [where] the information is too much."*
Information is determined by patients.	*"I think that [a] little information can be a dangerous thing, [and] too much information can be overwhelming. I ask the patients how much they want to know, and see what kind of questions they are asking."*
Structure of the IC process.	*"I'm not doing it like a,b,c. It depends on the case. If [a] person wants to know about side effects I tell him... I have a paper that I designed for [myself] and it reminds me what to ask, but it is individual... I would like to see more uniform[ity] in our profession. [Where] every patient that comes to acupuncture will get the same disclosures. A standard consent form ..."*

Table 4. Disclosure of Risks

Theme	Example
Elaboration on risks might be detrimental to the patients' healing response.	*"There are no risks [in what I do], but I do tell them symptoms can get worse, but only if they ask. I would never disclose a lot of risks. If I were to tell them, uncomfortable emotions may be released, they would never lay down on the table. I take away their opportunity... I think, that especially if it is their first treatment, they are nervous enough, so I don't want to make them more nervous."*
Disclosure of risks selectively based either on how common they are or how serious the consequences might be.	*"I tell [patients] the most common risks. If something is credibly safe, I wouldn't mention any risk. When I'm talking about most risky I'm talking about the severity. It's so severe so even if it's one to a million, people want to know about it."*
Uncertainty with respect to the body of knowledge about risks.	*"I think the difficult [part] is the fact that people have questions that I don't know fully how to answer. Sometimes I can't answer as people like... In many treatments we don't know all the [risks and benefits] because there are not many studies."*

Table 5. Potential Benefits

Theme	Example
Tailoring the information and potential benefits based on the nature of the clientele.	*"I believe that people [who] come to me have a big background about who I am and what I do. They want this treatment. Some patients come to me because someone told them [about the benefits] [but] they don't know anything about [what I do]…I'll ask them what they know about me, and about what I do and then I ask them what I want to know and my expectations. I never promise a cure. They don't sign anything but I let them know that I will answer every question in any time. If they trust me they will be willing to try it."*

Table 6. Discussions of Alternatives

Theme	Example
Lack of “enough” knowledge as a barrier.	*“We talk about alternatives, [but it] depends on the case. I'm unfamiliar with homeopathy for example…We talk also about conventional treatments… I'm trying to be fair... I definitely recommend other alternative providers if I think it would help patients, but only after I have tried my modality. It doesn't make sense to list alternatives up front.”*
